# Demographics, Presenting Features, and Outcomes of Adult Patients with Ocular Trauma

**DOI:** 10.1155/2024/8871776

**Published:** 2024-06-12

**Authors:** Leanne M. Clevenger, Jessica L. Cao, Megan S. Steinkerchner, Amy S. Nowacki, Alex Yuan

**Affiliations:** ^1^Cole Eye Institute, Cleveland Clinic, 9500 Euclid Avenue I-13, Cleveland 44195, OH, USA; ^2^The Retina Partners, 16500 Ventura Blvd Suite 250, Encino 91436, CA, USA; ^3^Quantitative Health Sciences, Lerner Research Institute, Cleveland Clinic, 9500 Euclid Avenue NB21, Cleveland 44196, OH, USA

## Abstract

**Introduction:**

Ocular trauma is a common cause of permanent vision loss in adults. The combination of an accurate clinical examination and imaging offers the best prognostic indicators for patients and helps to navigate treatment modalities. This is a retrospective chart review of examination and imaging findings for ocular trauma and how they correlate with treatment course and visual acuity (VA) outcomes.

**Methods:**

Adult patients with ocular trauma presenting to a single institution between January 2013 and December 2020 were evaluated. Initial examination and imaging findings were compared for associations with each other and with VA outcomes.

**Results:**

136 ocular traumas on 134 patients were included. The median presenting logMAR VA was 2.7 (interquartile range (IQR) 1.2–3.7) with 62% open globe injuries. The most commonly reported finding on initial CT scan was globe deformity (30%), on B-scan was choroidal detachment (20%), and on ultrasound biomicroscopy was intraocular foreign body, ciliochoroidal effusions, or angle recession (21% each). Worse vision was observed for patients positive for retinal detachment on initial B-scan compared to those negative for this finding at 6-month (median logMAR 2.7 vs. 0.5; *P* < 0.0001) and at final post-injury evaluation (median logMAR 3.7 vs. 0.4; *P* < 0.0001). Similarly, worse VA was observed for patients with choroidal detachment on initial B-scan compared to those without this finding at 6-month (median logMAR 1.4 vs. 0.5; *P* = 0.002) and at final post-injury evaluation (median logMAR 2.0 vs. 0.4; *P* < 0.0001). If positive conjunctiva/sclera examination findings were identified, 66% had positive findings on B-scan, whereas if the conjunctiva/sclera examination findings were absent, 41% had positive findings on B-scan (*P* = 0.005). If anterior chamber (AC) examination findings were positive, 59% had positive findings on B-scan, whereas if the AC examination findings were absent, 37% had positive findings on B-scan (*P* = 0.03). *Discussion*. The predictive value of examination findings in this study may offer insight as to long-term visual prognosis. Positive B-scan or CT findings should increase suspicion for open globe injuries.

## 1. Introduction

Ocular trauma is a common cause of permanent loss of vision in the adult population [1, 2]. According to a review by the World Health Organization, estimated 55 million injuries occur each year globally across all age groups, with 750,000 requiring hospitalization for these injuries [2]. In an analysis of the United States Eye Injury Registry, no less than 27% of patients with severe eye injury had final visual outcomes meeting the criteria for legal blindness (less than 20/200 vision) [1].

According to the Birmingham Eye Trauma Terminology (BETT) system, ocular trauma can be grouped as open (secondary to blunt rupture or lacerations) or closed (secondary to contusions or lamellar lacerations) [3]. At the time of patient presentation, final visual outcome can be predicted using the Ocular Trauma Score (OTS), which utilizes indicators such as initial visual acuity (VA), presence of rupture or perforating injury, endophthalmitis, retinal detachment, and an afferent pupillary defect (APD) [4]. Open globe injuries are associated with worse final visual outcome as well as higher economic burden [2, 5–8].

Ocular examination is often limited in the setting of ocular trauma, particularly in those patients with open globe injuries. Fundus examination is often limited secondary to corneal trauma and media opacities including vitreous hemorrhage. Ancillary imaging can provide more detailed information in the setting of ocular trauma. In particular, computed tomography (CT) imaging is instrumental in the evaluation of open globes. In the setting of ocular trauma, the sensitivity and specificity of CT scans in detecting open globe injuries have been reported to be as high as 75% and 93%, respectively [9]. CT scans are particularly useful to evaluate intraocular foreign bodies (IOFBs), which are found in 18–41% of open globe injuries [10–13]. A study by Patel et al. demonstrated that while clinical examination identified 45.6% of IOFBs at presentation, CT scan of the orbits was successful in identifying 94.9% of such cases [11]. B-scan imaging and ultrasound biomicroscopy (UBM) can also assist in characterizing anterior and posterior segment pathology in the setting of ocular trauma. UBM can elicit additional findings such as cyclodialysis or angle recession [14, 15]. B-scan ultrasonography can provide information regarding potential globe rupture, IOFBs, lens dislocation, vitreous hemorrhage, membranes, retinal tears, and retinal or choroidal detachments [16]. These imaging modalities are frequently applied in the setting of ocular trauma, and if urgent surgical repair is indicated, the opportunity for improved surgical planning is provided.

With the advancement of pars plana vitrectomy (PPV), final functional outcome in some traumatic cases has improved [1]. A review of the United States Eye Injury Registry (USEIR) demonstrated that 60.5% of posttraumatic eyes had improved VA after surgery, 30.5% experienced no change in vision, and 9% of patients had worsening of their final VA [17]. PPV has been implemented in the early removal of intraocular foreign bodies (IOFBs), clearing of vitreous hemorrhage, repair of complex retinal detachments, and treatment of endophthalmitis [18]. However, patients may require a variable number of operations, with a small percentage still developing phthisis following surgical intervention, particularly in those patients initially presenting with ocular rupture [19]. In a retrospective study of 113 patients who underwent PPV for ocular trauma, 7.3% of patients developed phthisis; half of the phthisical patients had presented with ocular rupture [19]. Indicators such as a relative APD, poor preoperative VA, retinal detachment, scleral laceration beyond the rectus muscle insertion, traumatic cataract, hyphema, and loss of vitreous have been identified as contributors to poor postoperative VA [6, 20].

While imaging is typically considered to be ancillary to the clinical examination in the evaluation of a patient with ocular trauma, the combination of the two offers the best prognostic indicators for patients. Associations between examination findings, imaging results, and visual outcomes could offer insight while navigating treatment modalities and surgical planning. The authors propose this retrospective chart review of examination and imaging findings at presentation and how they correlate with treatment course and final visual outcomes in these patients.

## 2. Methods

### 2.1. Patient Population

A retrospective chart review was performed on patients with a clinical diagnosis of ocular trauma presenting to the Cleveland Clinic Cole Eye Institute in Cleveland, Ohio, USA, between January 2013 and December 2020. Informed consent for inclusion in the study was waived due to the minimal risk to patient safety and confidentiality. The Cleveland Clinic Institutional Review Board approved retrospective data collection for this study, which was in accordance with the Declaration of Helsinki and Health Insurance Portability and Accountability Act. Patients at least 18 years of age presenting for their initial ophthalmologic assessment in the setting of blunt, penetrating, and/or perforating ocular trauma were included in the retrospective study. Inclusion criteria for this dataset required having an ocular imaging modality (B-scan ultrasonography or UBM) ordered. Imaging information was collected at the time of presentation or at initial examination post globe repair. Patients with pre-existing ocular disease, previous ocular trauma, and/or an initial ophthalmic exam at an outside institution were excluded from further review in the study.

It is notable that for most patients, urgent globe closure is performed and then B-scan or UBM imaging is performed at time of initial follow-up examination. This is valuable for prognostication as well as for presurgical planning if additional intervention is indicated (e.g., retinal detachment repair). UBM is contraindicated in the presence of an open globe with decreased intraocular pressure, as it will always slightly compress the globe in a very low-pressure eye. If the pressure is normal in the eye with suspected globe trauma, then UBM may be feasible. At our institution, B-scan is at times performed on eyes with suspected globe trauma and low intraocular pressure as long as particular precautions are followed by trained ophthalmic ultrasonographers. This includes scanning only through the lid with a copious amount of sterile ophthalmic gel. The gel is pooled over the eyelids and the probe “floats” on the surface of the gel. The probe should add very minimal to no pressure on the globe. Our ultrasonographers estimate that the pressure placed on the globe by the gel with probe is 0-1 mmHg. The globe does not change the shape while the B-scan is performed while floating on the pool.

### 2.2. Data Collection

Clinical data including demographic information, mechanism of ocular injury, ophthalmic examination findings, trauma-related imaging results, and ocular surgical procedures were collected for all patients from the electronic medical record (EMR). Demographic data comprised of patient age at presentation of ocular injury, gender, and ethnicity. The logMAR equivalent to Snellen VA, intraocular pressure, if obtained, and zone of ocular injury from the initial assessment were recorded for each patient. Zone of ocular injury was defined according to the location of the most posterior full-thickness aspect of the globe opening for open globe injuries or most posterior identified pathology for closed-globe injuries: Zone I limited to the cornea or corneoscleral limbus, Zone II involving the anterior 5 mm of the sclera, or Zone III at a location more than 5 mm posterior to the corneoscleral limbus [21]. The collection of relevant slit lamp exam and fundus ophthalmoscopy findings on initial ocular exam included, but was not limited to, subconjunctival hemorrhage, scleral laceration, corneal laceration, hyphema, angle recession, iridodialysis, lens capsular violation, lens subluxation or dislocation, vitreous hemorrhage, retinal tear and/or detachment, choroidal rupture, and optic nerve changes. CT scans, B-scan ultrasonography, and UBM findings in relation to the ocular trauma were recorded for the study population. The logMAR equivalent of Snellen visual acuity at the 6-month post-injury and final ophthalmologic clinic visit were recorded. Patients with no-light-perception (NLP) vision were assigned a logMAR score of 4.700, logMAR 3.700 for light perception (LP) vision, logMAR 2.700 for hand motion (HM) vision, and logMAR 1.400 for counting fingers (CF) vision [22]. Whether the patient underwent immediate surgical intervention at the time of ocular injury and information on any subsequent eye surgeries was collected.

### 2.3. Main Outcome Measures

The primary study outcomes were the relative incidence of open versus closed globe ocular trauma occurring at our institution and the relation between type of injury and ocular findings at initial ophthalmologic examination. In addition, we explored the relationship between ocular examination findings, imaging results, logMAR VA in the setting of globe trauma at 6-month post-injury and at the final post-injury evaluation, and requirement of immediate or delayed surgery. The 6-month logMAR VA was restricted to be a measurement occurring between 119 and 241 days post-injury (representing 6 months ± 2 months). The final post-injury evaluation logMAR VA was the VA obtained at the last recorded visit.

### 2.4. Statistical Analysis

Patient characteristics and zone of ocular injury are summarized with descriptive statistics including count (%) and median (IQR = *Q*_1_–*Q*_3_ = interquartile range between the first and third quartiles). Visual acuity measured by logMAR scores at both 6-month and the final post-injury evaluation were compared among groups, positive and negative, for various examination or imaging findings with Wilcoxon rank-sum tests. The proportion of open globe injuries was compared among groups, positive and negative, for various examination or imaging findings with chi-square or Fisher's exact tests. Patients were categorized according to their CT scan findings, and the proportion of those patients with positive examination findings was compared for various anatomical locations with chi-square or Fisher's exact tests. In addition, patients were categorized according to their examination findings, and the proportion of those which had positive imaging findings (B-scan and UBM) was compared with chi-square or Fisher's exact tests. A significance level of 5% was utilized. All analyses were conducted with JMP Pro version 16.1.0 (SAS Institute, Cary, NC) statistical software.

## 3. Results

### 3.1. Population Characteristics

A total of 136 unique ocular traumas on 134 patients were included in this investigation. Ninety-seven eyes of males (71%) and 39 eyes of female (29%) patients were analyzed. Ages ranged from 18 to 96 years, with a median of 55 years (IQR = 39–71). Population demographics and presenting clinical characteristics can be found in Table 1.

The median presenting logMAR visual acuity was 2.7, with an interquartile range (IQR) of 1.2 to 3.7. Eighty-four injuries (62%) were determined to have open globe injury at the time of initial presentation, all of which required immediate surgical repair. The majority of open globes were secondary to blunt rupture (46 eyes, 34%), penetrating lacerations (27 eyes, 20%), and intraocular foreign bodies (11 eyes, 8%). The majority of patients with closed globe injuries were secondary to ocular contusions (47 eyes, 35%), with a small percentage due to lamellar lacerations (3 eyes, 2%) or blunt rupture (2 eyes, 1%). Most injuries were located in Zone III (51 eyes, 38%), followed by Zone II (44 eyes, 32%) and then Zone 1 (41 eyes, 30%) injuries (Table 1).

Positive examination and imaging findings on presentation or immediately following initial repair are shown in Table 2. The most common examination findings in each anatomical subcategory were eyelid ecchymosis and edema (44 and 43 eyes, 32% and 32%, respectively), conjunctiva-subconjunctival hemorrhage (57, 42%), corneal-laceration (43, 32%), anterior chamber-hyphema (76, 56%), iris-iris prolapse (25, 18%), lens-traumatic cataract (17, 13%), vitreous-hemorrhage (26, 19%), optic nerve-optic neuropathy (4, 3%), macula-commotio (7, 5%), and periphery-retinal detachment (5, 4%). Of the 90 injuries where the patient received a CT scan surrounding the time of initial injury, the most common positive imaging finding was globe deformity (27, 30%). B-scans were obtained for 132 injuries, and the most common positive finding was choroidal detachment (26, 20%). Fourteen patients underwent UBM, and the most common positive findings were IOFB, ciliochoroidal effusions, or angle recession (3 each, 21%).

In addition to presenting visual acuity, logMAR visual acuity was recorded for 102 injuries (75%) at the 6-month post-injury time point. The time between injury and final post-injury evaluation for all patients ranged from 1 day to 7.8 years, with a median of 1.2 years (IQR = 0.4–3.0 years). The median logMAR visual acuity at the 6-month post-injury time point was 0.70 (IQR = 0.3–1.4) and at the final post-injury evaluation was 0.5 (IQR = 0.2 to 2.7). Within 6 months or by the final post-injury evaluation, 31 eyes had required additional surgeries beyond initial globe repair and 3 eyes required enucleation.

### 3.2. Association of Examination and Imaging Findings with Visual Acuity Outcomes

**Figure 1:**
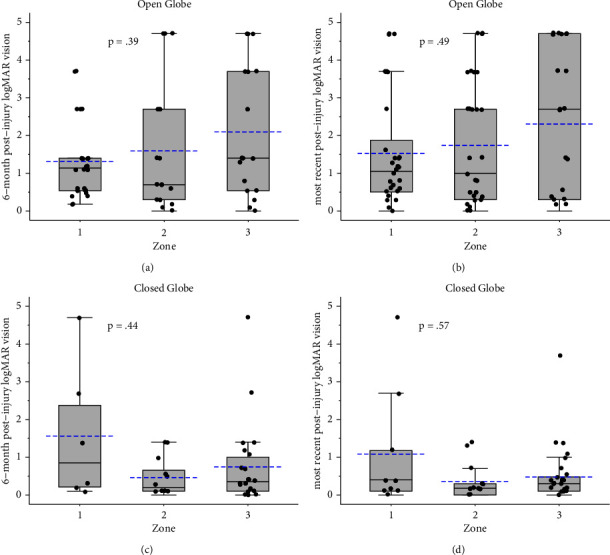
Six-month post-injury and final post-injury evaluation logMAR vision by injury zone and type box and Whisker's plots summarizing the distribution of logMAR scores by zone of injury at 6-month and final post-injury visit for open and closed globe injuries. The horizontal solid black line within the box represents the median while the bottom and top of the box represent the first and third quartiles. The horizontal dashed blue line indicates the group mean.

There was no significant difference in VA at 6-month or final postoperative evaluation between patients with different zones of injury, regardless of open or closed globe status (Figure 1). However, in the open globe category, there was a general trend towards worse vision with more posterior zones of injury (zones 2 and 3). The presence of positive examination findings (defined in Table 2) in the conjunctiva/sclera (compared to no findings) was associated with worse VA at 6-month (*P* = 0.04) and final post-injury (*P* = 0.003) evaluation. The presence of positive examination findings in the optic nerve (compared to no findings) was associated with worse VA at 6-month (*P* = 0.02) and final post-injury (*P* = 0.02) evaluation (Table 3). The presence of examination findings in the vitreous (compared to no findings) was associated with better VA at 6-month (*P* = 0.047) and final post-injury (*P* = 0.02) evaluation. The presence of findings in the periphery (compared to no findings) was associated with better VA at the final post-injury evaluation (*P* = 0.02), but this was not statistically significant at the 6-month post-injury time point (*P* = 0.49). The other examination findings were not significantly associated with vision at 6-month or final post-injury evaluation (Table 3).

Positive findings on initial B-scan (compared to negative, or absent, findings) were associated with worse VA at 6-month (*P*=0.04) and final post-injury (*P*=0.004) evaluation. More specifically, there was significantly worse vision in patients initially determined on B-scan to have a retinal detachment (compared to absence of retinal detachment) (median logMAR 2.7 vs. 0.5; *P* < 0.0001) or patients initially determined on B-scan to have a choroidal detachment (compared to absence of choroidal detachment) (median logMAR 1.4 vs. 0.5; *P*=0.03) at the 6-month post-injury. Results were similar at the final post-injury evaluation (median logMAR 3.7 vs. 0.4; *P* < 0.0001 for retinal detachment vs. no retinal detachment and median logMAR 1.4 vs. 0.4; *P*=0.01 for choroidal detachment vs. no choroidal detachment). Patients positive for membranes or linear sheet-like structures on B-scan were found to have worse vision than those with negative findings at the 6-month post-injury (median logMAR 4.2 vs. 0.7; *P*=0.04), but this was only marginally significant at the final post-injury (median logMAR 3.0 vs. 0.5; *P*=0.07) evaluation. Patients positive for vitreous hemorrhage on B-scan were found to have better vision than those without these findings at 6-month post-injury (median logMAR 0.3 vs 1.0; *P*=0.03) and final post-injury (median logMAR 0.2 vs. 0.7; *P*=0.04) evaluation. Patients positive for an intraocular foreign body on B-scan had better vision than those negative for these findings at 6 months (median logMAR 0 vs. 0.8; *P*=0.004), but this was not significant at the final post-injury evaluation (median logMAR 0.2 vs. 0.5; *P*=0.12). Differences in VA outcomes were not observed when comparing patients with positive findings on CT scan to those negative for findings at 6-month (*P*=0.26) nor at the final post-injury (*P*=0.20) evaluation. Similarly, differences in VA outcomes were not observed when comparing patients with positive findings on UBM to those with negative (or absent) findings at 6-month (*P*=0.43) nor at the final post-injury (*P*=0.78) (Table 3) evaluation.

### 3.3. Association of Examination and Imaging Findings with Type of Injury

The proportion of patients presenting with open globe injuries was investigated, as they are related to different examination or imaging findings (Table 4). Patients with positive corneal examination findings were more likely to have open globe injuries (positive findings = 72% open globe; negative findings = 45% open globe; *P*=0.002), which was largely driven by examination findings of corneal laceration (positive findings = 93% open globe; negative findings = 47% open globe; *P* < 0.0001) and dehisced penetrating keratoplasty (PKP) or keratoprosthesis (KPro) (positive findings = 100% open globe; negative findings = 58% open globe; *P*=0.007). Patients presenting with corneal abrasion (positive = 85% open globe; negative = 65% open globe; *P*=0.05) or corneal edema (positive = 43% open globe; negative = 65% open globe; *P*=0.06) showed marginally different rates of open globe injuries. Those patients with positive macular examination findings were less likely to present with open globe injuries (positive = 0% open globe; negative = 65% open globe; *P*=0.001). No other examination component had a statistically significant association with type of injury.

Patients with positive CT scan findings were more likely to have open globe injuries (positive = 83% open globe; negative = 55% open globe; *P*=0.005). More specifically, the presence of an IOFB (positive = 94% open globe; negative = 65% open globe; *P*=0.02) or globe deformity (positive = 85% open globe; negative = 65% open globe; *P*=0.08) on CT scan was more commonly open globe injuries. Patients with positive B-scan findings more frequently had open globe injuries (positive findings = 74% open globe; negative findings = 48% open globe; *P*=0.002), particularly for those patients with reported choroidal detachment (positive = 83% open globe, negative = 54% open globe; *P*=0.002), retinal detachment (positive = 87% open globe, negative = 58% open globe; *P*=0.009). Positive versus negative UBM findings did not significantly differ in the type of presenting injury (*P*=0.99).

### 3.4. Association among Examination and Imaging Findings

We examined the agreement of imaging findings with examination findings. The proportion of positive B-scan findings on acquired imaging was compared among patients with a positive versus negative examination finding. Examination findings were not associated with positive CT or UBM findings. Positive conjunctiva/sclera examination findings were associated with positive findings on B-scan (positive examination findings = 66% positive B-scan; absent examination findings = 41% positive B-scan; *P*=0.005). More specifically, patients with subconjunctival hemorrhage on examination were more likely to have choroidal detachment on B-scan than without this finding (positive examination findings = 44% choroidal detachment; absent examination findings = 21% choroidal detachment; *P*=0.007; Table 5). Membranes were more likely to be seen on the B-scan of patients with scleral laceration observed on the examination (positive = 29% membranes; negative = 0% membranes; *P* < 0.0001) or patients with uveal prolapse observed on examination (positive = 50% membranes; negative = 0% membranes; *P* < 0.0001). Pathology in the anterior chamber examination was associated with positive findings overall on B-scan (positive examination findings = 59% positive B-scan; negative examination findings = 37% positive B-scan; *P*=0.03). In particular, patients presenting with a hyphema on examination compared to without were more likely to have a dislocated native or implanted intraocular lens seen on B-scan (positive examination findings = 11% dislocated lens/IOL; negative examination findings = 0% dislocated lens/IOL; *P*=0.01). Patients with a shallow or flat anterior chamber compared to without were more likely to have choroidal detachment (positive examination findings = 56% choroidal detachment; negative examination findings = 27% choroidal detachment; *P*=0.02) (Table 5).

## 4. Discussion

The classification of ophthalmic injury as determined by clinical examination is imperative in the initial assessment of ocular trauma. We found that 62% of the cases reviewed presented with open globe injuries, and that location of injury was slightly more common in Zone III (38%) although evenly spread across Zones I and II (30% and 32%, respectively). Madan AH et al. found that in a pediatric population, 64% of patients with globe injuries were identified as having open globe injuries, with the majority of all patients having Zone I injuries [23]. In a geriatric population, open globe injuries are more commonly seen in Zones II and III as compared to younger patients and are less frequently associated with IOFBs [24]. Our study varies from general trends in the literature in that a statistically significant difference between visual acuity outcomes and presenting zone of injury was not identified for either closed or open globe injuries [25–27]. There was a trend towards better final visual acuity in patients with Zone I open globe injuries, although this was not statistically significant (Figures 1(a) and 1(b)). Similarly, a trend for better visual acuity outcomes in Zones II and III closed-globe injuries was seen although not statistically significant (Figures 1(c) and 1(d)).

While all patients with open globe injuries required initial surgical repair, it is important to note that a significant number of patients (31 eyes, 23%) required additional surgery during the follow-up period. Examples of subsequent posttraumatic surgical repair include secondary surgery for traumatic cataract, retinal detachment, traumatic glaucoma, or other anterior segment reconstructive surgeries. In a retrospective study of patients presenting with open globe injury, Dulz et al. revealed that almost half of patients (49%) presenting with Zone III injuries underwent additional surgical intervention for posttraumatic retinal detachment [28].

The predictive value of examination findings in this study may offer insight as to a long-term visual prognosis. Positive examination findings in the conjunctiva/sclera category (compared to negative findings) were associated with worse logMAR VA at follow-up for reviewed patients (median logMar 1.2 with findings vs 0.5 without; *P*=0.04). The worse visual acuity outcomes for patients with positive conjunctiva/sclera examination findings (compared to negative findings) were largely driven by those patients with subconjunctival hemorrhage (median logMAR 1.1 vs. 0.4; *P*=0.02). While conjunctiva/sclera findings as a whole were not associated with a higher proportion of open globe injuries, the presence of a subconjunctival hemorrhage on examination was associated with choroidal detachment on B-scan (positive examination findings = 44% choroidal detachment; negative examination findings = 21% choroidal detachment; *P*=0.007). The authors would suggest that the presence of subconjunctival hemorrhage should prompt thorough examination or imaging to rule out posterior segment involvement, but its absence does not exclude posterior segment involvement. Furthermore, positive B-scan or CT findings should increase suspicion for open globe injuries, prompting possible globe exploration. Positive examination findings in the optic nerve category were also associated with worse visual acuity outcomes. Traumatic optic neuropathy is known to produce long-standing detrimental effects on visual acuity, with no clearly beneficial treatment options [29].

Better VA outcomes were noted for those patients positive for a vitreous hemorrhage (compared with negative findings for this) on examination at 6-month (median logMAR 0.5 with positive finding vs. 0.8 negative findings; *P*=0.047) and at final post-injury (median logMAR 0.3 positive finding vs 0.7 negative findings; *P*=0.02) evaluation. Prior studies have determined that vitreous hemorrhage is associated with the worse visual prognosis in patients with largely Zone II injuries; however, as positive findings for vitreous hemorrhage was not subcategorized according to the zone for this analysis, the authors propose that the visualization and documentation of vitreous hemorrhage in this study imply the lack of corneal or other anterior segment pathology (such as dense hyphema) which would obstruct view and could more readily be addressed or repaired with subsequent surgery [30]. Similarly, having an IOFB on initial examination in the present study was associated with better visual acuity at 6 months (median logMAR 0 positive for IOFB vs. 0.8 negative for IOFB; *P*=0.004). Visualization of an IOFB on initial examination suggests the presence of an adequate view through the anterior segment (i.e., the absence of findings such as a total hyphema or lens/cornea disruption) and more localized posterior pathology which is likely to be improved with surgery.

It is of interest that the visual acuity was better at 6-month post-injury than at the final visit for those patients with vitreous hemorrhage on presentation. There is potential for formation of cataract (induced by trauma or by subsequent pars plana vitrectomy) at final follow-up which could cause worse vision at final follow-up than at 6 months. In addition, there is likely a component of selection bias as those patients with more significant visual deficits could continue presenting for ophthalmic care, whereas those patients with better vision are discharged from care. Both possibilities are beyond the current analysis of this study and present as potential limitations.

In the setting of trauma, CT scan is adjunctive to clinical examination when evaluating for pathology including orbital fractures or foreign bodies, retrobulbar hemorrhage, globe integrity, or intraocular foreign bodies. It is a helpful tool, but not sensitive enough to rule out open globe injury alone [9]. Eyelid, cornea, and lens findings on examination were more frequently associated with positive CT scan findings in this study. CT scans positive for an IOFB or globe deformity were more commonly associated with open globe injuries (*P*=0.02 and *P*=0.08, respectively). Joseph et al. determined that patients who had a poor final VA (VA <2/200, Snellen: 20/2000) or who underwent enucleation had significantly more CT findings than patients with good final VA, indicating that CT scans may have some predictive values [9]. However, in the present study, positive findings on CT imaging were not associated with a difference in VA at either follow-up point (*P*=0.26 at 6-month and *P*=0.20 at final post-injury evaluation).

In the ophthalmic population, B-scan imaging is routinely utilized to evaluate the globe when the view to the fundus is obstructed by anterior segment pathology or other media opacities. These same factors likely influence long-term visual acuity as found in this population; unsurprisingly, positive findings on B-scan were associated with worse VA at follow-up (*P*=0.04 at 6-month and *P*=0.004 at final post-injury evaluation). Retinal and choroidal detachments on B-scan were more commonly found in patients with open globe injuries at the time of presentation (*P*=0.009 and *P*=0.002, respectively). Patients with subconjunctival hemorrhage on initial examination were more likely to have choroidal detachment seen on B-scan (*P*=0.007). Those with scleral laceration or uveal prolapse on examination were more likely to have membranes on B-scan (*P* < 0.001 for each). This is unsurprising as violation of the sclera or uvea indicates a more serious injury that likely prompts a robust intraocular inflammatory response that leads to membrane formation. Thus, patients presenting with these findings should undergo a serious conversation with their ophthalmologists regarding the possible development of vision-threatening membranes. Patients with hyphema were more likely to have a dislocated native or implanted lens on B-scan (*P*=0.01). A shallow or flat anterior chamber was associated with choroidal detachment (*P*=0.02). Knowledge of the status of the posterior segment structures is critical for surgical planning, and B-scan imaging often provides insights to optimize outcomes. As the utility of UBM is largely to investigate the organization of anterior segment structures or to evaluate for a retained IOFB, it is not surprising that positive findings here were not correlated with an open globe injury or final visual acuity in this population.

Potential limitations to this study include its status as a retrospective cohort study, relying on past documentation and nonstandardized follow-up schedule. In addition, the clinical skills of the examining ophthalmologist varied widely, as the study included patients initially examined by clinical residents and fellows at various stages of their training. This might have had an impact on the exam documented as well as imaging modalities that were obtained and interpreted. The relatively small number of patients undergoing certain imaging modalities could potentially bias conclusions, while decreasing the statistical power for respective imaging investigations. In addition, 25% of patients did not have a 6-month follow-up visit, which limits the conclusions one can draw regarding visual acuity at this point. However, almost all patients (98%) were included in the final post-injury evaluation analysis, which is why we present analysis of both time points. Unfortunately, the final post-injury evaluation also had a large range of 1 day to 7.8 years. Another limitation is the inherent selection bias for patients who have undergone ancillary imaging following trauma. These patients tend to have difficult exams or exam elements that are not assessable (i.e., posterior segment exam in a patient with 8-ball hyphema). These patients also tend to have more severe initial injury compared to patients who do not require ancillary testing.

## 5. Conclusions

These results suggest that while examination findings are imperative in the initial classification of ocular trauma, specific examination findings are associated with positive findings on ancillary imaging which may aid in surgical planning and help predict the visual prognosis. Further investigation with a prospective study where all patients receive the same ancillary tests would be needed to determine if specific imaging findings are truly predictive of final outcomes and an eventual need for secondary surgery.

## Figures and Tables

**Table 1:** Clinical and demographic characteristics of the study population.

Characteristic	Overall
Number of eye injuries	136
Number of patients	134
Age (years)	55 (39–71) min = 18, max = 96
Male	97 (71%)
Race	
White	93 (69%)
Black	39 (29%)
Others	2 (2%)
Presenting vision (logMAR)	2.7 (1.2–3.7)
Presenting intraocular pressure (IOP)^*∗*^	17 (10–40)
Type of injury	
Open	84 (62%)
Closed	52 (38%)
Mechanism of injury	
Open-blunt rupture	46 (34%)
Closed contusion	47 (35%)
Open penetrating laceration	27 (20%)
Open intraocular foreign body	11 (8%)
Closed lamellar laceration	3 (2%)
Closed blunt rupture	2 (1%)
Open perforating laceration	0 (0%)
Zone of injury	
1: the whole cornea, including corneoscleral limbus	41 (30%)
2: corneoscleral limbus to a point 5 mm posterior into the sclera	44 (32%)
3: posterior to the anterior 5 mm of the sclera	51 (38%)
Imaging obtained	
Computed tomography (CT) scan	90 (66%)
B-scan	132 (97%)
Ultrasound biomicroscopy (UBM)	14 (10%)
Immediate operative repair required^*∗∗*^	84 (62%)

Descriptive statistics are reported as either count (%) or median (*Q*_1_–*Q*_3_). ^*∗*^Missing for 61 injuries. ^*∗∗*^Including immediate postoperative period if urgent surgery is required.

**Table 2:** Examination and imaging findings investigated.

Anatomical region	Examination findings investigated
Eyelid	Ecchymosis, edema, and laceration
Conjunctiva/sclera	Subconjunctival hemorrhage, conjunctival laceration/abrasion, scleral laceration, and uveal prolapse
Cornea	Corneal laceration, corneal edema, corneal abrasion, uveal prolapse, dehisced PK graft, and dehisced keratoprosthesis
Anterior chamber	Hyphema, cell/flare, shallow depth, and flat depth
Iris	Iris prolapse, traumatic mydriasis, iridodialysis, and peaked pupil
Lens	Lens dislocation, IOL dislocation, traumatic cataract, phacodinesis, and capsule disruption
Vitreous	Vitreous hemorrhage and IOFB
Optic nerve	Traumatic optic neuropathy
Macula	Commotio
Periphery	Commotio, retinal detachment, and preretinal hemorrhage

Imaging modality	Imaging findings investigated

CT scan	Orbital fracture, IOFB, globe deformity, and lens/IOL dislocation
B-scan	RD, IOFB, vitreous hemorrhage, dislocated lens/IOL, choroidal detachment, and membranes
UBM	Ciliochoroidal effusion, angle recession, IOFB, cyclodialysis, and iris/ciliary body rotation

PK: penetrating keratoplasty, IOFB: intraocular foreign body, IOL: intraocular lens, CT: computed tomography, RD: retinal detachment, and UBM: ultrasound biomicroscopy.

**Table 3:** Associations among examination and imaging findings and post-injury logMAR visual acuity.

		6-month post-injury evaluation			Final post-injury evaluation		
		*N*	logMAR	*P* value	*N*	logMAR	*P* value
*Examination findings*							
Lid	Positive	52	1.1 [0.3–2.4]	0.23	73	0.7 [0.2–2.7]	0.26
	Negative	50	0.6 [0.2–1.4]		60	0.5 [0.1–1.4]	
Conjunctiva/sclera	Positive	44	1.2 [0.3–3.5]	**0.04**	64	1.1 [0.2–3.7]	**0.003**
	Negative	58	0.5 [0.2–1.4]		69	0.4 [0.1–1.2]	
Cornea	Positive	68	1.1 [0.2–1.4]	0.19	81	0.8 [0.2–2.7]	0.08
	Negative	34	0.5 [0.3–1.3]		52	0.3 [0.2–1.4]	
Anterior chamber	Positive	78	0.7 [0.3–1.4]	0.82	98	0.5 [0.2–2.7]	0.83
	Negative	24	0.7 [0.1–1.4]		35	0.5 [0.2–1.4]	
Iris	Positive	38	0.7 [0.2–1.4]	0.49	48	0.4 [0.1–2.4]	0.31
	Negative	64	0.7 [0.3–2.7]		85	0.5 [0.2–2.7]	
Lens	Positive	28	0.5 [0.3–1.3]	0.23	33	0.4 [0.0–1.3]	0.10
	Negative	74	1.0 [0.2–2.7]		100	0.7 [0.2–2.7]	
Vitreous	Positive	20	0.5 [0.0–1.3]	**0.047**	25	0.3 [0.0–1.0]	**0.02**
	Negative	82	0.8 [0.3–1.7]		108	0.7 [0.2–2.7]	
Optic nerve	Positive	4	3.1 [1.4–4.7]	**0.02**	4	3.2 [2.7–4.5]	**0.02**
	Negative	98	0.7 [0.2–1.4]		129	0.5 [0.2–1.4]	
Macula	Positive	4	0.2 [0.0–1.0]	0.11	7	0.2 [0.1–0.4]	0.08
	Negative	98	0.7 [0.3–1.4]		126	0.6 [0.2–2.7]	
Periphery	Positive	8	0.7 [0.1–1.2]	0.49	10	0.1 [0.0–0.4]	**0.02**
	Negative	94	0.7 [0.3–1.4]		123	0.6 [0.2–2.7]	
*Imaging findings*							
CT scan	Positive	39	1.1 [0.4–1.4]	0.26	51	0.8 [0.3–3.7]	0.20
	Negative	25	0.7 [0.1–2.1]		36	0.5 [0.3–1.4]	
B-scan	Positive	57	1.1 [0.3–2.7]	**0.04**	69	1.1 [0.3–3.7]	**0.004**
	Negative	43	0.5 [0.1–1.4]		60	0.4 [0.1–1.2]	
UBM	Positive	10	0.4 [0.1–1.1]	0.43	11	0.4 [0.1–1.4]	0.78
	Negative	5	0.8 [0.2–3.7]		5	0.3 [0.1–4.2]	

Reported as median (*Q*_1_–*Q*_3_) = interquartile range (first quartile to the third quartile). *P* values result from Wilcoxon rank-sum tests. CT: computed tomography and UBM: ultrasound biomicroscopy. The bold numbers indicate statistically significant *P* values.

**Table 4:** Associations among examination and imaging findings and type of trauma.

Findings	Positive findings	*N*	Proportion of open globe injuries (%)	*P* value
*Examination findings*				
Lid	Yes	75	57	0.24
	No	61	67	
Conjunctiva/sclera	Yes	66	65	0.43
	No	70	59	
Cornea	Yes	83	72	**0.002**
	No	53	45	
Anterior chamber	Yes	100	62	0.93
	No	36	61	
Iris	Yes	48	67	0.39
	No	88	59	
Lens	Yes	33	70	0.28
	No	103	59	
Vitreous	Yes	26	46	0.07
	No	110	65	
Optic nerve	Yes	4	75	0.99^F^
	No	132	61	
Macula	Yes	7	0	0.001^**F**^
	No	129	65	
Periphery	Yes	10	40	0.18^F^
	No	126	63	
*Imaging*				
CT scan	Yes	52	83	**0.005**
	No	38	55	
B-scan	Yes	70	74	**0.002**
	No	66	48	
UBM	Yes	11	45	0.99^F^
	No	5	40	

*P* values result from chi-square tests unless indicated as a Fisher's exact test^F^. Bold values are statistically significant *P* values.

**Table 5:** Individual components of associations among conjunctiva/sclera and B-scan findings and anterior chamber and B-scan findings.

Examination findings	Positive B-scan findings					
	Retinal detachment	Intraocular foreign bodies	Vitreous hemorrhage	Dislocated lens/IOL	Choroidal detachment	Membranes
Conjunctiva/sclera (positive vs. negative)						
Subconjunctival hemorrhage	24% vs. 13% 0.16	5% vs. 6% 0.99	7% vs. 5% 0.44	2% vs. 9% 0.14	44% vs. 21% **0.007**	5% vs. 1% 0.31
Conjunctival laceration/abrasion	12% vs. 18% 0.99	12% vs. 6% 0.40	25% vs. 5% 0.08	12% vs. 6% 0.40	12% vs. 31% 0.43	0% vs. 3% 0.99
Scleral laceration	21% vs. 17% 0.71	14% vs. 5% 0.20	14% vs. 5% 0.20	0% vs. 7% 0.60	36% vs. 30% 0.76	29% vs. 0% **<0.0001**
Uveal prolapse	0% vs. 19% 0.35	0% vs. 6% 0.99	12% vs. 6% 0.40	0% vs. 6% 0.99	25% vs. 31% 0.99	50% vs. 0% **<0.0001**
Anterior chamber (positive vs. negative)						
Hyphema	20% vs. 14% 0.36	5% vs. 7% 0.73	8% vs. 3% 0.47	11% vs. 0% **0.01**	34% vs. 26% 0.35	4% vs. 2% 0.63
Cell/flare	14% vs. 18% 0.99	14% vs. 5% 0.20	7% vs. 6% 0.99	0% vs. 7% 0.60	14% vs. 32% 0.23	0% vs. 3% 0.99
Shallow or flat	25% vs. 16% 0.48	0% vs. 7% 0.59	0% vs. 7% 0.59	0% vs. 7% 0.60	56% vs. 27% **0.02**	0% vs. 3% 0.99

Data reported are the proportion with positive imaging B-scan findings among those with positive examination findings versus those with negative (or absent) examination findings. *P* values result from Fisher's exact tests. Bold values are statistically significant *P* values.

## Data Availability

Access to data is restricted due to the policies of the institutional review board of the Cleveland Clinic Foundation.
